# DNA methylation-based measures of biological aging and cognitive decline over 16-years: preliminary longitudinal findings in midlife

**DOI:** 10.18632/aging.204376

**Published:** 2022-11-11

**Authors:** Rebecca G. Reed, Judith E. Carroll, Anna L. Marsland, Stephen B. Manuck

**Affiliations:** 1 Department of Psychology, Dietrich School of Arts and Sciences, University of Pittsburgh, Pittsburgh, PA 15260, USA; 2 Cousins Center for Psychoneuroimmunology, Department of Psychiatry and Biobehavioral Science, Jane and Terry Semel Institute for Neuroscience and Human Behavior, David Geffen School of Medicine, University of California, Los Angeles, CA 90095, USA

**Keywords:** epigenetic age, aging biomarker, pace of aging, geroscience, cognitive aging

## Abstract

DNA methylation-based (DNAm) measures of biological aging associate with increased risk of morbidity and mortality, but their links with cognitive decline are less established. This study examined changes over a 16-year interval in epigenetic clocks (the traditional and principal components [PC]-based Horvath, Hannum, PhenoAge, GrimAge) and pace of aging measures (Dunedin PoAm, Dunedin PACE) in 48 midlife adults enrolled in the longitudinal arm of the Adult Health and Behavior project (56% Female, baseline Age_M_ = 44.7 years), selected for discrepant cognitive trajectories. Cognitive Decliners (*N* = 24) were selected based on declines in a composite score derived from neuropsychological tests and matched with participants who did not show any decline, Maintainers (*N* = 24). Multilevel models with repeated DNAm measures within person tested the main effects of time, group, and group by time interactions. DNAm measures significantly increased over time generally consistent with elapsed time between study visits. There were also group differences: overall, Cognitive Decliners had an older PC-GrimAge and faster pace of aging (Dunedin PoAm, Dunedin PACE) than Cognitive Maintainers. There were no significant group by time interactions, suggesting accelerated epigenetic aging in Decliners remained constant over time. Older PC-GrimAge and faster pace of aging may be particularly sensitive to cognitive decline in midlife.

## INTRODUCTION

People age chronologically at the same rate but show substantial individual differences in their rates of biological aging, or the gradual, multi-system decline in physiology that occurs with aging. Recent advances are underway to quantify biological aging. DNA methylation (DNAm)-based measures, including first- and second-generation epigenetic clocks and pace of aging measures, are promising aging biomarkers that predict morbidity and mortality independent of chronological age [1–3].

Links between DNAm measures and risk for cognitive decline have been less well characterized, despite the substantial and growing burden of cognitive decline and dementia [4]. The majority of existing evidence on DNAm measures and neuropsychologically assessed cognitive function is cross-sectional [5] and cannot address whether changes in biological aging are associated with changes in cognition. Four studies to date [6–9] have examined but did not find changes in cognitive function relating to changes in first- or second-generation epigenetic clocks.

First-generation clocks, including Horvath [10] and Hannum [11], were trained to predict chronological age. Therefore, Horvath and Hannum clocks exhibit high correlations with chronological age; however, they predict morbidity and mortality more weakly than second-generation clocks [12, 13]. Second-generation clocks, including PhenoAge [14] and GrimAge [12], were optimized for lifespan prediction. Specifically, PhenoAge and GrimAge were developed to capture DNAm patterns that not only change with chronological age, but also account for differences in risk for morbidity and mortality. Finally, the latest DNAm measures include epigenetic “pace of aging” metrics [2, 3] and principal components (PC)-based clocks [15]. Pace of aging measures differ from first- and second-generation clocks in that they were trained to predict longitudinal changes in multi-system biomarkers [2, 3]. Specifically, Dunedin PoAm was trained in individuals of the same chronological age to predict changes in 18 biomarkers across 12 years (age 26 to 38), and Dunedin PACE, an updated version, was trained to predict changes in 19 biomarkers across 20 years (age 26 to 45) [2, 3]. Last, PC-based clocks were developed to enhance the reliability of traditional epigenetic clocks (Horvath, Hannum, PhenoAge, and GrimAge), which use individual CpG sites that are noisy and unreliable [16]. Instead, PC-based clocks use principal components (shared systematic variation across many CpG sites) rather than individual CpGs to estimate PC-clock ages [15] (see Supplementary Materials for additional DNAm clock descriptions). These latest DNAm measures (Dunedin PoAm, Dunedin PACE, and PC-clocks) may be particularly robust predictors of cognitive decline, but these associations have yet to be thoroughly examined, including longitudinally.

This preliminary study examined overall levels and changes in traditional and PC-based first- and second-generation epigenetic clocks and pace of aging measures in participants selected from a larger prospective cohort to represent extremes of maintained and declining cognitive function (termed Maintainers and Decliners, respectively) between a baseline visit when participants were in midlife and a second visit approximately 16 years later. We hypothesized that overall, cognitive Decliners would be biologically older compared to cognitive Maintainers. We also explored whether cognitive Decliners would show faster biological aging (i.e., steeper increases in DNAm over time) compared to cognitive Maintainers; and whether particular cognitive domains associated more strongly than others with measures of biological aging. We expected that PC-based clocks of enhanced reliability would outperform traditional clocks and that second-generation clocks and pace of aging measures trained to predict morbidity, mortality, and multi-system decline would outperform first-generation clocks optimized for age prediction. Notably, we tested several DNAm measures because a comparative analysis approach is recommended to simultaneously evaluate the utility of many DNAm measures and determine which ones are associated with aging outcomes of interest [17].

## RESULTS

Neuropsychological tests were administered and biological age was estimated at both time 1 (T1) and time 2 (T2) for 24 people who declined in cognitive function (Decliners) and 24 who maintained cognitive function (Maintainers) from T1 to T2 (mean years between assessments = 15.9, range: 15.4 to 16.9), selected using an extreme groups approach (see Methods). Table 1 summarizes study participant characteristics. Decliners and Maintainers did not significantly differ on chronological age, sex, education, race, body mass index, smoking status, or T1 cognition (a composite score derived from neuropsychological tests for spatial reasoning, working memory, processing speed, executive function, and attention; see Methods). Decliners’ cognitive composite decreased from T1 to T2 (T1_M_ = 67.61; T2_M_ = 53.89, *p* < 0.001) whereas Maintainers’ cognitive composite did not change over time (T1_M_ = 66.48; T2_M_ = 67.56, *p* = .189). The observed cognitive decline was more than a standard deviation decline, a clinically noticeable change in cognitive performance associated with risk for future cognitive impairments. Normative values on several neuropsychological tests were further examined to contextualize changes in the cognitive composite. As the sample performed above average at T1, the Decliners’ change can be interpreted as moving from above average to average, whereas the Maintainers remained slightly above average at both time points (see Supplementary Results). All individuals in the Decliner and Maintainer groups denied being diagnosed with dementia. Adjudications were not performed, so clinical determinations regarding mild cognitive impairment (MCI) cannot be made.

**Table 1:** Characteristics of cognitive decliners (*n* = 24) and maintainers (*n* = 24).

	**Total**	**Decliners**	**Maintainers**	***p*-value^a^**
Age (yrs), mean (SD)				
T1	44.79 (6.34)	44.57 (6.43)	45.01 (6.38)	0.815
T2	60.67 (6.27)	60.42 (6.34)	60.91 (6.32)	0.791
Education (yrs), mean (SD)				
T1	15.15 (2.45)	14.46 (2.38)	15.83 (2.37)	0.051
T2	15.46 (2.71)	14.79 (2.54)	16.12 (2.77)	0.089
Sex, *N* (%)				
Male	21 (43.8)	11 (45.8)	10 (41.7)	0.239
Female	27 (56.2)	13 (54.2)	14 (58.3)	
Race, *N* (%)				
White	44 (91.7)	22 (91.7)	22 (91.7)	0.656
Black or African American	4 (8.3)	2 (8.3)	2 (8.3)	
Time between T1 and T2, mean (SD)	15.87 (0.33)	15.85 (0.27)	15.90 (0.39)	0.592
BMI (kg/m^2^), mean (SD)				
T1	24.70 (3.60)	25.13 (3.37)	24.27 (3.85)	0.411
T2	26.80 (5.18)	27.79 (5.35)	25.85 (4.93)	0.203
Current smoker, N (%)				
T1, No	36 (75.0)	19 (79.2)	17 (70.8)	0.107
T1, Yes	12 (25.0)	5 (20.8)	7 (29.2)	
T2, No	39 (81.2)	19 (79.2)	20 (83.3)	0.261
T2, Yes	9 (18.8)	5 (20.8)	4 (16.7)	
Cognitive Composite, mean (SD)				
T1	67.05 (8.60)	67.61 (9.17)	66.48 (8.15)	0.653
T2	60.73 (11.19)	53.89 (10.05)	67.56 (7.58)	**<0.001**

Total sample size is 48. Means and standard deviations (SD) are displayed for continuous measures; sample size (*N*) and percentages (%) are shown otherwise. T1: time 1; T2: time 2. ^a^*p*-value comparing groups. Dependent *t*-tests were used for continuous variables; chi-square tests were used for categorical variables. *p*-values are bold if <0.05.

**Figure 1:**
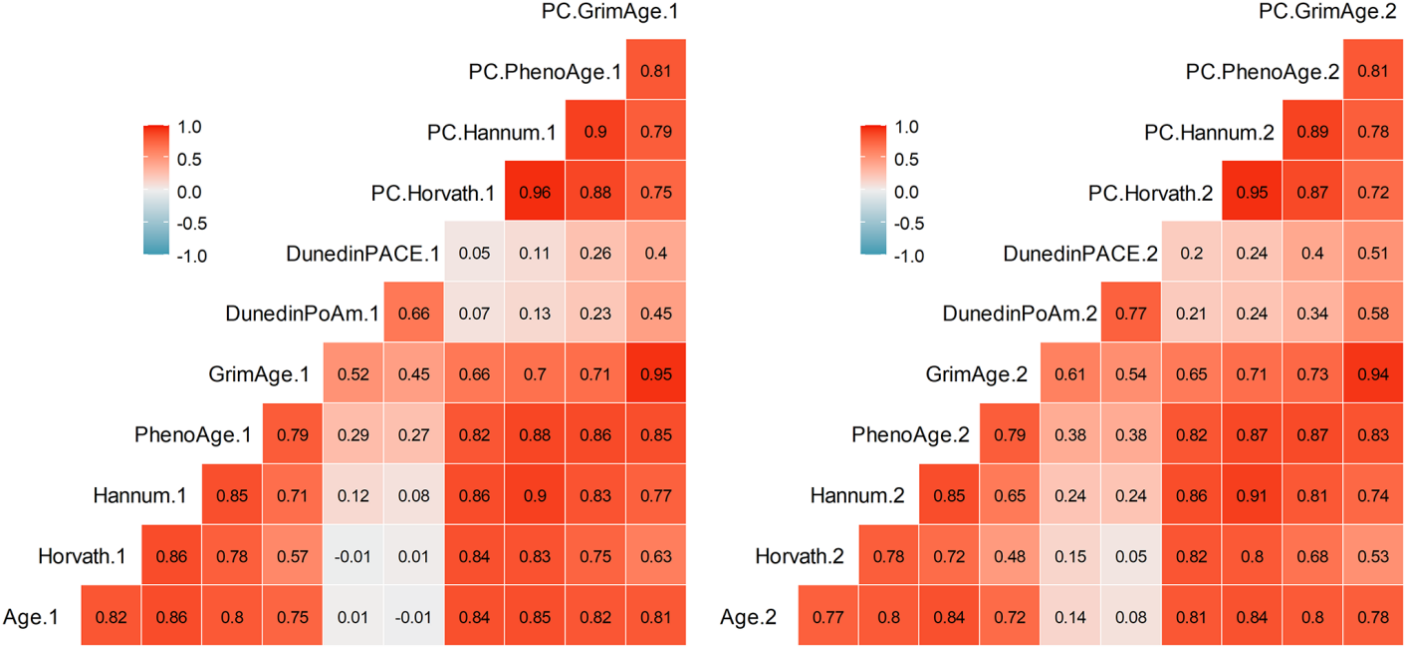
**Pearson correlations among DNAm measures at Time 1 (left) and Time 2 (right).** Correlations greater than *r* = .29 are statistically significant at *p* < .05.

**Figure 2:**
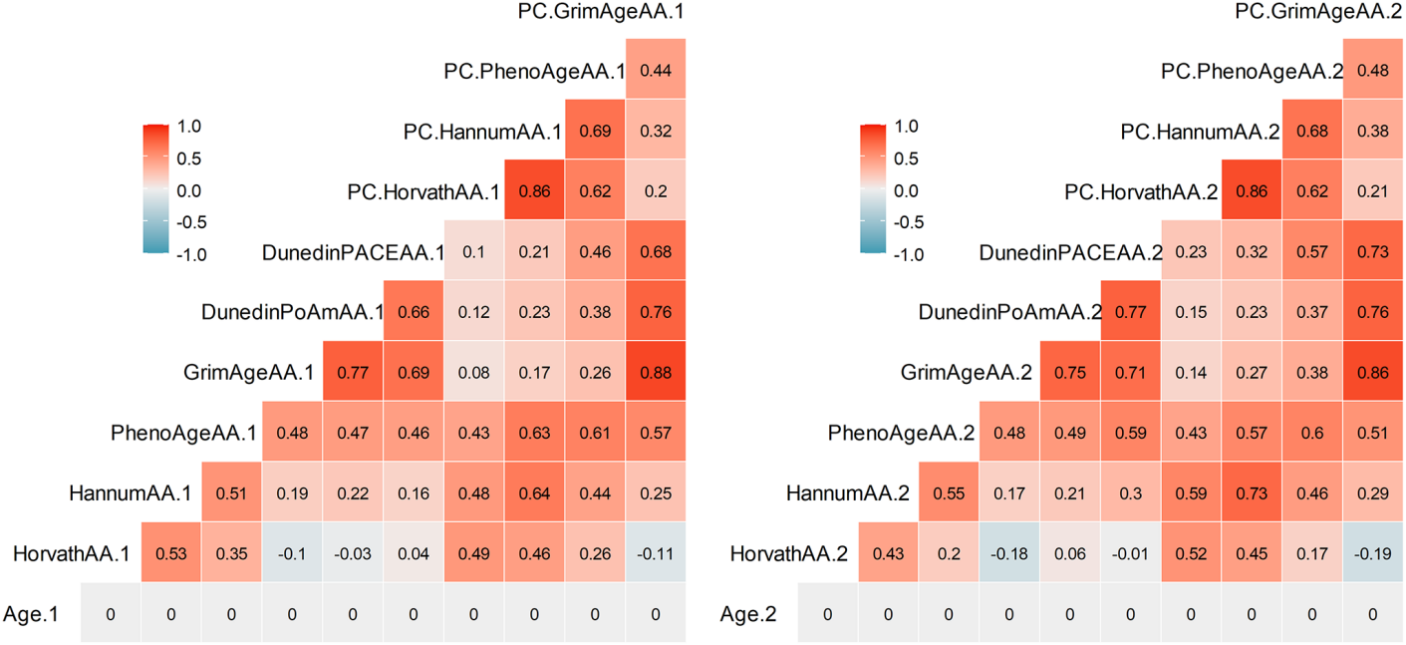
**Pearson correlations among DNAm measures independent of chronological age (denoted Age Acceleration, AA) at Time 1 (left) and Time 2 (right).** Correlations greater than *r* = .29 are statistically significant at *p* < .05.

Table 2 summarizes descriptive statistics for the DNAm measures: Horvath, Hannum, PhenoAge, GrimAge, Dunedin PoAm, Dunedin PACE, PC-Horvath, PC-Hannum, PC-PhenoAge, and PC-GrimAge (see Methods). All DNAm measures exhibited rank-order stability between baseline and follow-up (*r*’s ranged from 0.71 to 0.93); GrimAge and PC-GrimAge had the highest test-retest correlations (both *r* = .93) and Dunedin PACE (*r* = .73) and Dunedin PoAm (*r* = .71) were lower. In addition, there were strong and similar inter-correlations among DNAm measures within each time point (Figure 1). The exceptions were Dunedin PoAm and Dunedin PACE, which only correlated with each other (*r* = .66–.77) and with GrimAge (*r* = 0.45–0.61) and PC-GrimAge (*r* = .40–.58) at T1 and T2. DNAm measures independent of chronological age (denoted Age Acceleration, AA) are displayed in Figure 2. As compared to raw DNAm measures, the inter-correlations among DNAmAA measures were smaller within each time point, with the exception of Dunedin PoAm-AA and Dunedin PACE-AA, which were more strongly correlated with GrimAgeAA (*r* = .69–.77) and PC-GrimAgeAA (*r* = .68–.76), as well as with PhenoAgeAA (*r* = .46–.59) and PC-PhenoAgeAA (*r* = .37–.57) at T1 and T2.

**Table 2:** Descriptive statistics among the DNAm measures.

	**T1, M (SD)**	**T2, M (SD)**	**Change per year, M (SD)**	**Test-retest (*r*)**
Chronological Age	44.79 (6.34)	60.67 (6.27)	1.00 (0.00)	1.00
Horvath	46.32 (6.52)	59.07 (6.44)	0.80 (0.19)	0.89
Hannum	37.27 (6.75)	50.38 (6.69)	0.83 (0.18)	0.91
PhenoAge	34.46 (8.15)	49.76 (9.03)	0.96 (0.31)	0.85
GrimAge	48.43 (6.68)	60.86 (7.07)	0.78 (0.16)	0.93
Dunedin PoAm	1.01 (0.08)	1.04 (0.08)	0.002 (0.004)	0.71
Dunedin PACE	0.91 (0.12)	0.97 (0.13)	0.003 (0.006)	0.73
PC-Horvath	46.77 (6.25)	58.49 (6.26)	0.74 (0.15)	0.93
PC-Hannum	52.97 (6.38)	65.40 (6.30)	0.78 (0.18	0.90
PC-PhenoAge	44.07 (8.23)	59.19 (8.05)	0.95 (0.29)	0.85
PC-GrimAge	58.09 (5.97)	70.98 (6.30)	0.81 (0.15)	0.93

Means (M) and standard deviations (SD) are shown for Time 1 (T1) and Time 2 (T2) DNAm measures. Change per year represents the average rate of change in each DNAm measure per year. Test-retest correlations are displayed as Pearson *r*.

### Time and group main and interacting effects on DNAm

**Figure 3:**
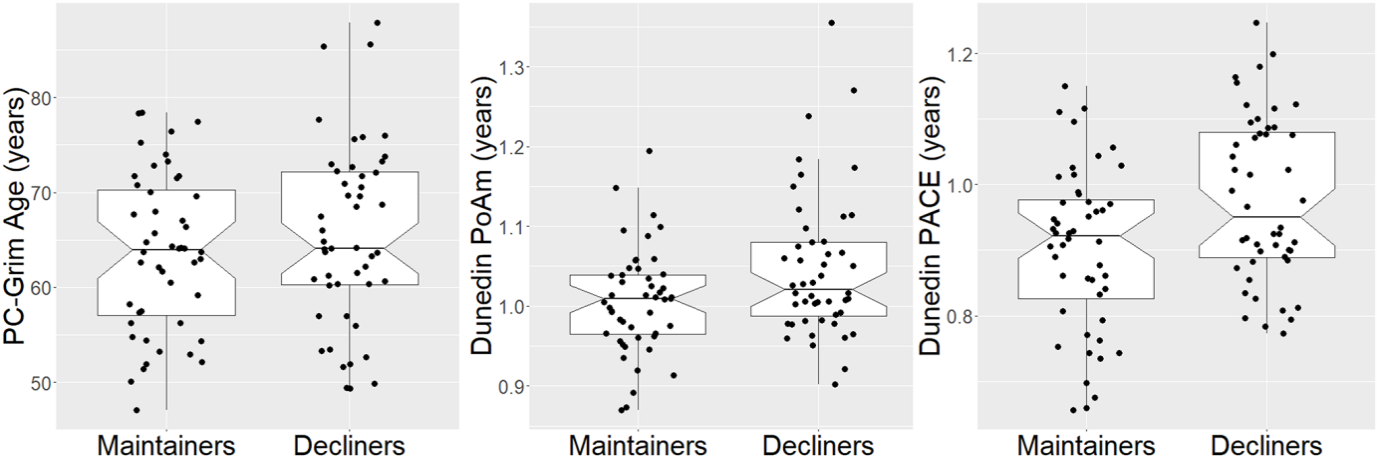
**Boxplots of significant group effects on PC-GrimAge, dunedin PoAm, and dunedin PACE.** Two values are shown per person, but analyses accounted for repeated measures within person.

The traditional and PC-based epigenetic clocks and pace of aging measures significantly increased over time, generally consistent with or underestimating the time elapsed between study visits (Table 3 and Supplementary Table 1). With respect to group differences, Decliners overall had an older PC-GrimAge (γ = 2.05, SE = .94, *t*(44) = 2.18, *p* = .035) and a faster pace of aging on both Dunedin PoAm (γ = .042, SE = .021, *t*(44) = 2.06, *p* = .045) and Dunedin PACE (γ = .075, SE = .033, *t*(44) = 2.28, *p* = .027) than Maintainers (Table 3, Figure 3). (Decliners did not significantly differ from Maintainers on PC-PhenoAge (γ = 1.22, SE = 1.20, *t*(44) = 1.02, *p* = .31)). In other words, Decliners were on average 2.05 years older than Maintainers using PC-GrimAge; in terms of pace of aging, Decliners biologically aged at rates .042 (Dunedin PoAm) and .075 (Dunedin PACE) faster than Maintainers. For example, if Maintainers age at a rate of 1.0 biological year per chronological year, Decliners age at 1.042 (Dunedin PoAm) and 1.075 (Dunedin PACE) biological years per chronological year. In analyses that adjusted for multiple comparisons using the Benjamini-Hochberg correction [18] (see Data Analyses), these group differences remained statistically significant at a false discovery rate (FDR) of .10 but not .05. In addition, in sensitivity analyses that further controlled for percentages of CD8 T cells, CD4 T cells, NK cells, plasma blasts, monocytes, and granulocytes, these group differences remained statistically significant (Supplementary Table 2). Furthermore, results were similar from logistic regression models that regressed Cognitive Decliner group membership (1) [vs. Cognitive Maintainer (0)] on average biological age, controlling for sex and baseline chronological age: a 1-year increase in PC-GrimAge was associated with a .22 increased log-odds of being in the Cognitive Decliner group (*p* = .049); in addition, a 1-year rate increase in Dunedin PoAm and Dunedin PACE were associated with 9.91 (*p* = .061) and 6.03 (*p* = .034) increased log odds of being in the Cognitive Decliner group. The Dunedin PoAm finding is no longer statistically significant likely due to power loss moving from a multilevel modeling framework to logistic regression. In the main analyses, there were no group by time interactions (*p*s > .24).

**Table 3:** Main effects of group and time on PC-Clocks and pace of aging measures.

***Predictors***	**PC-Horvath**		**PC-Hannum**		**PC-PhenoAge**		**PC-GrimAge**		**Dunedin PoAm**		**Dunedin PACE**	
	***γ (CI)***	***p***	***γ (CI)***	***p***	***γ (CI)***	***p***	***γ (CI)***	***p***	***γ (CI)***	***p***	***γ (CI)***	***p***
Intercept	48.78 (47.22–50.35)	**<0.001**	54.46 (52.90–56.02)	**<0.001**	44.14 (41.88–46.40)	**<0.001**	58.14 (56.41–59.87)	**<0.001**	0.98 (0.95–1.02)	**<0.001**	0.88 (0.82–0.94)	**<0.001**
Female	−2.90 (−4.58–−1.22)	**0.002**	−2.26 (−3.91–−0.61)	**0.010**	−1.21 (−3.59–1.17)	0.323	−1.91 (−3.77–−0.05)	0.050	0.01 (−0.03–0.05)	0.714	−0.00 (−0.07–0.06)	0.914
Baseline Age	0.83 (0.70–0.96)	**<0.001**	0.85 (0.72–0.98)	**<0.001**	1.05 (0.86–1.24)	**<0.001**	0.78 (0.64–0.93)	**<0.001**	0.00 (−0.00–0.00)	0.528	0.00 (−0.00–0.01)	0.672
Group-*Decliners*	−0.77 (−2.43–0.90)	0.372	−0.43 (−2.07–1.21)	0.608	1.22 (−1.14–3.58)	0.315	2.05 (0.20–3.90)	**0.035**	0.04 (0.00–0.08)	**0.045**	0.08 (0.01–0.14)	**0.027**
Time	11.72 (11.04–12.39)	**<0.001**	12.42 (11.60–13.24)	**<0.001**	15.12 (13.82–16.41)	**<0.001**	12.89 (12.23–13.55)	**<0.001**	0.03 (0.02–0.05)	**0.001**	0.05 (0.03–0.08)	**<0.001**

95% Confidence Intervals (CI) are reported.

### Exploring specific cognitive components on DNAm

To further explore whether the several components of cognitive functioning associated differentially with PC-GrimAge and pace of aging measures, we conducted secondary analyses using the same adjusted multilevel model predicting T1 and T2 DNAm, but instead of the categorical Group predictor, we tested the continuous scaled version of each cognitive component at T2 to determine which cognition component(s) were significantly associated with DNAm-based measures of biological aging. We focused on T2 cognitive components because this was the time point that differentiated the two groups (see Supplementary Table 3).

Results are depicted in Table 4. In terms of executive function, worse performance on T2 Trail A-B was significantly associated with older PC-GrimAge (*p* = .013) and faster pace of aging for Dunedin PoAm (*p* = .016) and for Dunedin PACE (*p* = .019). In addition, worse performance on Stroop Color-Word was significantly associated with older PC-GrimAge (*p* = .017). In terms of processing speed, slower Trail A and worse performance on Stroop Word were associated with faster pace of aging for Dunedin PACE (Trail A: *p* = .046, Stroop Word: *p* =.035). Finally, in terms of spatial reasoning, worse matrix reasoning was associated with faster pace of aging for Dunedin PACE (*p* = .041). The following components of cognition at T2 were not significantly associated with DNAm: working memory (Digit Span forward and backward), attention (Digit Vigilance), and one measure of processing speed (Stroop Word). In analyses that adjusted for multiple comparisons, only the associations with the executive function tests remained statistically significant at an FDR of .05 or .1 (Trail A-B: all *p*_adj_ = .019; Stroop Color-Word: *p*_adj_ = .051). Results further adjusted for cell percentages did not differ and are in Supplementary Table 4.

**Table 4:** Main effects of scaled Time 2 cognitive components on PC-GrimAge and pace of aging measures.

	**PC-GrimAge**		**Dunedin PoAm**		**Dunedin PACE**	
	***γ (CI)***	***p***	***γ (CI)***	***p***	***γ (CI)***	***p***
Matrix Reasoning	−0.049 (−0.120–0.021)	0.179	−0.001 (−0.002–0.001)	0.467	−0.003 (−0.005–−0.000)	**0.041**
DS-Forward	−0.026 (−0.074–0.022)	0.286	−0.000 (−0.001–0.001)	0.601	−0.001 (−0.003–0.001)	0.231
DS-Backward	−0.035 (−0.086–0.015)	0.180	−0.000 (−0.001–0.001)	0.521	−0.001 (−0.003–0.001)	0.220
Trail A	−0.163 (−0.365–0.039)	0.120	−0.004 (−0.008–0.001)	0.092	−0.007 (−0.014–−0.000)	**0.046**
Trail A-B	−0.097 (−0.171–−0.024)	**0.013**	−0.002 (−0.004–−0.000)	**0.016**	−0.003 (−0.006–−0.001)	**0.019**
Stroop Word	−0.054 (−0.113–0.005)	0.079	−0.001 (−0.002–0.000)	0.122	−0.002 (−0.004–−0.000)	**0.035**
Stroop Color	−0.057 (−0.115–0.002)	0.065	−0.001 (−0.002–0.000)	0.093	−0.001 (−0.003–0.001)	0.236
Stroop Color-Word	−0.068 (−0.121–−0.014)	**0.017**	−0.001 (−0.002–0.000)	0.089	−0.002 (−0.004–0.000)	0.111
Digit Vigilance-pg1	−0.052 (−0.123–0.019)	0.162	−0.002 (−0.003–0.000)	0.117	−0.001 (−0.004–0.002)	0.414
Digit Vigilance-pg2	−0.063 (−0.138–0.012)	0.109	−0.002 (−0.004–0.000)	0.078	−0.002 (−0.005–0.001)	0.167

95% Confidence Intervals (CI) are reported. Models included female, baseline age, and time (estimates not shown). Higher scaled cognitive scores indicate better performance. Abbreviation: DS: digit span.

## DISCUSSION

This is the first report to explore changes over time in several of the latest DNAm biological aging measures – including traditional and PC-based epigenetic clocks and pace of aging measures – in an age-, race-, sex-, education-, cognition-, and body mass index- matched case control comparison and where cases were selected for having cognitive performance declines on objective neuropsychological tests. There were no group differences in DNAm slopes over time, which may be due to low statistical power, but is in line with the few previous studies that have examined only first- and second-generation epigenetic clocks [6–9]. However, cognitive decline was related to an overall older PC-GrimAge and a faster pace of aging (Dunedin PoAm and Dunedin PACE) compared to those without cognitive decline over this 16-year time frame. These group differences remained statistically significant when corrected for multiple comparisons at a false discovery rate of .10.

There was no evidence of associations between the first-generation epigenetic clocks and cognitive decline. Rather, our findings point to the second-generation clock PC-GrimAge as being more sensitive to cognitive change, which aligns with others who report associations between GrimAge, but not Horvath or Hannum, and worse cognitive performance cross-sectionally [19], worse future cognitive performance [8], and cognitive decline from adolescence to age 45 [3] and from age 70 to 79 [20]. Notably, we did not observe associations with (PC)-PhenoAge and cognitive decline, which may be due to limited power, but is also consistent with other reports [3, 8]. Although PhenoAge and GrimAge are both second-generation clocks, they differ in how they were trained: PhenoAge was created by identifying CpGs that predict a composite measure of mortality-related blood biomarkers (see Supplementary Materials for biomarker list) and chronological age [14]. Conversely, GrimAge was created by generating DNAm surrogates of morbidity- and mortality-related plasma proteins (see Supplementary Materials) and smoking pack-years; then time-to-death was regressed onto these DNAm surrogates, chronological age, and sex to identify the CpGs [12]. The blood-based biomarkers across both epigenetic clocks reflect the functioning of similar physiological systems (e.g., immune, kidney, metabolic), but GrimAge also explicitly includes the effects of smoking, which is an established risk factor for cognitive decline and dementia [21]. In addition, of the first- and second-generation clocks, GrimAge and PC-GrimAge tend to have the highest reliability due to its two-step DNAm calculation [3, 15]; thus, this measurement property may also explain why GrimAge tends to outperform other clocks, including PhenoAge. However, these reasons remain speculative and future studies with DNAm data should continue to evaluate and report associations across multiple DNAm measures (including the newest pace of aging measures, below) to facilitate comparison across studies, reconcile inconsistencies, and facilitate their inclusion in future meta-analyses and systematic reviews.

In addition to PC-GrimAge, faster pace of aging was associated with cognitive decline. This report is the first to replicate Belsky and colleagues’ [2, 3] findings of Dunedin PoAm and Dunedin PACE associating with cognitive decline. Our findings suggest that pace of aging measures, which were developed from Dunedin Study participants aged 26–45, can inform cognitive outcomes in middle-aged and older adults. Pace of aging measures may be particularly sensitive to pre-clinical cognitive changes because they are indexed by a longitudinal panel of biomarkers across multiple physiological systems, which may more closely reflect the mechanisms of cognitive decline, relative to first-generation epigenetic clocks that are optimized for age prediction. Interestingly, the epigenetic clocks that pace of aging was most strongly correlated with at T1 and T2 were GrimAge and PC-GrimAge (Figures 1, 2), suggesting that these DNAm measures may be detecting some shared biological aging signals. A limitation to the current DNAm measures is a lack of mechanistic understanding of their underlying biology. Current work is underway to deconstruct these DNAm composite measures into distinct “modules” that may reflect functionally related biological changes [22]. Each epigenetic clock is comprised of differing proportions of CpGs from a given module; however, in line with our findings, GrimAge and DunedinPoAm share a similar composition of modules and have higher quantities of modules that are stronger predictors of morbidity and mortality, as compared to PhenoAge, Horvath, and Hannum [22]. Continued efforts to examine the underlying mechanisms of DNAm measures will aid our understanding of why certain clocks outperform others in predicting health outcomes, including cognitive health.

All DNAm measures significantly increased over time; however, these estimates of biological aging did not increase between T1 and T2 more steeply in Decliners, compared to Maintainers, as evidenced by the absence of a significant group by time interaction. In other words, DNAm estimates of biological aging were associated with the 16-year change in cognitive functioning, but did not progress more rapidly in Decliners than among Maintainers, which may suggest that Decliners’ accelerated profile of epigenetic aging was established prior to the initial assessment. However, we note that we had limited power to detect small and moderate effects (particularly interaction effects); therefore, we cannot confidently infer whether the non-significant group by time interactions are due to truly null effects and/or due to the smaller sample size.

In exploring whether particular cognitive domains may covary with PC-GrimAge and pace of aging measures more strongly than others, executive function showed the most consistent associations, as well as withstanding correction for multiple comparisons. One previous report links older epigenetic age estimated from other clocks, including Horvath’s intrinsic and Hannum derived extrinsic epigenetic age acceleration and PhenoAge, but not GrimAge, to poorer executive function in African Americans with HIV and a control group [23]; others report null associations between GrimAge and executive function composites [24, 25], and between Dunedin PACE and one test of executive function, Trails B [26]. Therefore, converging evidence for associations between DNAm and specific cognitive domains remains inconclusive. Future studies will benefit from investigating separate cognitive domains (in addition to general composites, which is more commonly done), to shed light on which components of cognition may be more or less affected.

The current study focused on neuropsychologically-assessed cognitive decline, which can indicate future risk for dementia [27]. Indeed, in other studies, DNAm measures predicted MCI and clinical diagnosis of Alzheimer’s Disease (e.g., [26, 28]). No participants in our sample reported having a dementia diagnosis, but adjudications were not performed, so MCI status could not be assessed. However, descriptively, the group with cognitive performance decrements over time experienced greater than a standard deviation change in their average composite score, an indication they may be at future cognitive risk, with their T2 assessments falling slightly below normative values on several neuropsychological tests (see Supplemental Results). It remains unclear whether these individuals will manifest future cognitive impairments, but this magnitude of decline is considered clinically meaningful [29].

Strengths of this study include the longitudinal design with a relatively long follow-up of 16 years; the comprehensive assessment of cognition across several domains known to decline with age; and the recommended analysis of multiple DNAm measures [17] that allowed for comparisons across traditional and PC-based epigenetic clocks and pace of aging measures. However, this preliminary study had limited power to detect small and moderate effects (particularly interaction effects), although we maximized our ability to detect effects by selecting cognitive groups from the tails or extremes of the distribution of cognitive change. In addition, the cognition composite approach used to identify Cognitive Decliners vs. Maintainers assumed that the neuropsychological tests have the same meaning and factor structure across the 16-year time frame in both groups; our smaller, multi-group sample does not meet sample size recommendations for testing measurement invariance [30, 31]. However, using a latent variable approach and testing measurement invariance is an important future direction for cognitive change research, and may yield stronger effects than a composite approach (e.g., [32]). Other limitations include only two time points for longitudinal analysis; limited generalizability in terms of education and race; and DNAm measured in blood but not the brain, although blood-brain global DNAm profiles are highly correlated (*r* = .86) [33].

In conclusion, these preliminary results suggest PC-GrimAge and DNAm based pace of aging measures (Dunedin PoAm and PACE) associate with 16-year, neuropsychologically-validated cognitive decline in midlife. The results warrant a larger-scale study to better examine longitudinal associations between changes in DNAm measures and changes across multiple cognitive domains. Ultimately, establishing DNAm measures as biomarkers of cognitive function in midlife may offer pre-clinical markers of a molecular aging mechanism that can help identify individuals at increased risk for cognitive impairment and dementia in later life.

## METHODS

### Participants

Participants were selected from a longitudinal arm of the Adult Health and Behavior (AHAB)-1 study, which comprises a registry of behavioral and biological measurements for the study of midlife individual differences [34]. AHAB-1 participants were first recruited at 30–54 years of age via mass-mail solicitation from southwestern Pennsylvania and were relatively healthy. Study exclusions at the time of initial recruitment (time 1) were a reported history of atherosclerotic cardiovascular disease, chronic kidney or liver disease, cancer treatment in the preceding year, and major neurological disorders, schizophrenia, or other psychotic illness. Other exclusions included pregnancy and reported use of insulin, glucocorticoid, antiarrhythmic, psychotropic, or prescription weight-loss medications. Baseline (T1) assessments occurred between 2001 and 2005 and follow-up (T2) assessments began in 2017 and are ongoing, with additional subjects being added at the time of writing.

### Selection of participant groups

Using an extreme groups approach, a subset of AHAB-1 participants was selected for the current study: 24 Cognitive Decliners (i.e., those who showed the most decline in cognition from T1 to T2 based on changes in a cognitive composite score, described below) and 24 matched Cognitive Maintainers (i.e., those who maintained cognitive composite levels from T1 to T2, matched to Decliners on demographics and health). The selection was carried out in the following steps: First, from the 300 available AHAB-1 participants with both T1 and T2 data who were enrolled for follow-up (T2) evaluation between June, 2017 and March, 2020, we excluded those who reported medical conditions having potential cognitive sequelae, as might be associated with Alzheimer’s disease, stroke, transient ischemic attack, multiple sclerosis, Parkinson’s disease, epilepsy, brain cancer, or brain cyst, and people who endorsed having a head injury, concussion, or spinal cord injury. We also excluded people with diagnosed diabetes or HbA1c greater than or equal to 7%; individuals who reported exposure in the previous 12 months to any of the neurocognitive tests administered here; were missing more than 3 of 10 cognitive measurements used in the present analyses; or for whom we lack a stored T1 blood sample sufficient for DNA extraction and DNAm profiling. These exclusions resulted in 167 remaining participants. From the 167, we selected the 24 most extreme cognitive decliners, identified using the cognitive composite (described below). Next, we identified the 50 most extreme cognitive maintainers, and from those 50, matched on sex, race, T1 age, T1 education, T1 cognitive composite, and T1 body mass index to obtain the matched 24 cognitive maintainers. One-to-one multivariate matching based on Mahalanobis distance was performed using the Match function in R (Matching package) [35]. Matching was performed without replacement and by randomly breaking ties. Groups (Decliners, Maintainers) were identified blind and prior to assessment of DNAm measures.

### Procedure

Sociodemographic, cognitive, psychosocial, and instrumented biological measurements were collected over multiple study visits at both T1 and T2. At T1, the neuropsychological tests used in the present analyses were administered at visit 1 and blood was drawn at visit 2. On average, there were 30.85 days between visits 1 and 2 for the sample analyzed (median = 25.5, range: 2 to 98). At T2, the neuropsychological tests used in the present analyses were administered at visits 2 and 3 and blood was drawn at visit 2. On average, there were 26.1 days between visits 2 and 3 for the sample analyzed (median = 16.5, range: 8 to 102). AHAB was approved by the University of Pittsburgh Institutional Review Board, and all participants provided written informed consent.

### Measures

#### Demographic and health characteristics

Self-reported sex, race, years of education, and smoking status were assessed. Measures of height and weight were obtained to determine body mass index (in kg/m^2^).

#### Cognition

T1 and T2 neuropsychological tests used in the present analyses capture several domains of cognitive function: spatial reasoning, working memory, visuomotor processing speed, executive function, and attention. A cognition composite was used (described below).

#### Spatial reasoning

The Matrix Reasoning subtest from the Wechsler Abbreviated Scale of Intelligence [36, 37] was used to assess spatial perception and reasoning. This test involves viewing an incomplete matrix and selecting the response option that completes the matrix. Higher scores correspond to better spatial reasoning.

#### Working memory

Working memory was assessed with the Digit Span subtest from the Wechsler Adult Intelligence Scale – III (WAIS-III) [37]. The participant is read sequences of numbers and is asked to recall the numbers in the same order (forward) or in reverse order (backward). Higher scores indicate better working memory.

#### Visuomotor processing speed

Participants completed the first parts of the Trail Making Test [38] and the Stroop Color-Word Test [39] to assess processing speed. Part A (in seconds) of the Trail Making Test requires participants to draw a line connecting circles numbered from 1 to 25 as quickly as possible. Higher scores correspond to poorer processing speed. The first two parts of the Stroop Color-Word Test require participants to (A) read aloud a list of color names (i.e., red, green, blue) printed in black ink and (B) name the colors of the inks (i.e., “XXXX” written in blue ink) as quickly as possible. Scores are the number of correct responses within a 45-second period, with higher scores indicating better performance.

#### Executive function

Participants were administered two tests of executive functioning: task switching on Part B of the Trail Making Test [38] and the interference score of the Stroop Color-Word Test [39]. The Trail Making Test Part B requires subjects to draw a line connecting numbered and lettered circles as quickly as possible, alternating between numbers and letters in ascending numerical and alphabetical order (e.g., 1-A-2-B-3-C…, etc.). To derive a measure of executive function relatively independent of psychomotor speed, time to completion of Part B is subtracted from Part A, such that higher scores indicate better performance. Assessing ability to resist cognitive interference, the Stroop Color-Word Test requires subjects to read aloud as quickly as possible from 3 pages of color word lists: pages 1 and 2 provide tests of processing speed, previously described. On Page 3 individuals are asked to report the color of the ink used to print the name of incongruent colors (e.g., “blue” for blue ink used to spell the color name “red”), thus requiring participants to inhibit a prepotent response (color word naming). Scores are the number of correct responses within a 45-second period, with higher scores indicating better performance.

#### Attention

Digit Vigilance pages 1 and 2 [40] was administered to assess vigilant visual tracking and capacity for sustained attention. This test requires participants to rapidly scan a page of numbers arrayed in rows and to cross out only digits designated as targets as quickly as possible. Time (in seconds) was recorded. Higher scores correspond to lower performance.

#### Cognition composite

A cognition composite was calculated using raw (not standardized or normed) test scores. First, the Trail Making Test Part A and Digit Vigilance Times were multiplied by (-1) so that higher scores correspond to better performance; then the proportion of maximum scaling approach [41] was applied to the individual subtests. This approach transforms each score to a metric from 0 (minimum observed) to 1 (maximum observed) by first transforming the score range from 0 to the highest observed value and then dividing by the highest observed value. The resulting value between 0 and 1 was multiplied by 100. This approach does not change the multivariate distribution and covariate matrix of the transformed variables and is the recommended approach for longitudinal data [42]. The scaled individual tests (Matrix Reasoning, Digit Span forward and backward, Trail Making Test A and A-B, Stroop word, Stroop color, and Stroop color-word, and Digit Vigilance pages 1 and 2) were averaged together to create a cognition composite using all available data. At T1, no cognition data were missing. At T2, 1 participant was missing the Stroop test and 19 were missing Digit Vigilance pages 1 and 2 and 1 was missing just page 2. Higher composite scores indicate better cognition. Notably, this composite approach assumes that the individual neuropsychological tests have the same meaning and factor structure over time. The composite’s multilevel reliability was calculated using coefficient omega (omegaSEM function in the multilevelTools package) and was adequate at both the between- (ω = .80, 95% CI [.62, .98]) and within-person levels (ω = .85, 95% CI [.79, .91]).

### Tissue acquisition and processing

Fasting blood was collected by a trained phlebotomist between 8:00am and 10:00am. Whole blood samples were frozen in −80°C until time of DNA extraction and analysis. DNA was extracted using the DNeasy Blood and Tissue Kit (Qiagen) at the UCLA Cousins Center for Psychoneuroimmunology. Purified DNA was concentrated using GeneJET PCR Purification Kit (ThermoFisher) and suspended in the elution buffer to a minimum of 12.5 ng/ul before plating in a 96-well plate. DNA was quantified using the Quant-iT dsDNA Assay Kit, high sensitivity (Invitrogen).

Consideration for variability across assay chips was addressed by organizing samples from the same individual to be placed together on the same chip but randomly assigned by ID. In addition, samples from Decliners and Maintainers were assured to be evenly distributed within each chip, and position within chip was randomized.

### DNA methylation data pre-processing

Bisulfite conversion using the Zymo EZ DNA Methylation Kit (ZymoResearch, Orange, CA, USA) and subsequent hybridization of the Human Methylation 850 K EPIC chip (Illumina, San Diego, CA, USA) and scanning (iScan, Illumina) were performed by the UCLA Neuroscience Genomics Core facilities according to the manufacturer’s protocols. DNA methylation image data were processed in R statistical software (version 4.1.1) using the *minfi* Bioconductor package (version 1.38.0) [43]. We checked for samples with >1% of sites with detection *p*-values >0.01 (*n* = 0) and for samples with DNA methylation predicted sex discordant with recorded sex (*n* = 0). The minfi preprocessNoob function was used to normalize dye bias and apply background correction before obtaining methylation beta-values.

### Epigenetic clocks and pace of aging measures

The following traditional first- and second-generation epigenetic clocks were estimated using available online software (http://dnamage.genetics.ucla.edu/new, with the “Normalize Data” and “Advanced Analysis” options selected for blood samples): Horvath (353 CpGs) [10], Hannum (71 CpGs) [11], PhenoAge (513 CpGs) [14], and GrimAge (1030 CpGs) [12]. Given the low reliability of existing epigenetic clocks [15], we used available R code that uses principal component (PC) analyses to improve reliability of epigenetic clocks and calculated the following “PC” clocks: PC-Horvath, PC-Hannum, PC-PhenoAge, and PC-GrimAge. Finally, we also calculated Dunedin pace of aging measures using available R code: DunedinPoAm (46 CpGs) [2] and DunedinPACE (173 CpGs) [3].

### Covariates

Analyses were adjusted for participant age and sex. Additionally, because DNAm profiles may differ between cell subtypes [44] and cell composition changes with age, the percentages of six cell subtypes (CD8 total, CD4 total, NK cells, plasma blasts, monocytes, and granulocytes) were estimated from Horvath’s website using the Houseman method [45] (and see [46] for validation) and further controlled for in sensitivity analyses. Some may consider controlling for cell subtypes to be unnecessary adjustment or overadjustment because cell subtypes may contribute to the observed differences in DNAm or be on a mediation pathway linking DNAm to aging outcomes; however, we present results both ways for interested readers.

### Data analysis

All analyses were conducted using the traditional and PC-based epigenetic clocks and pace of aging measures. Further mention of DNAm refers to all measures unless specified.

The DNAm measures were modeled individually in two multilevel models with repeated measures nested within person. Model 1 included the main effect of group (Maintainers, Decliners) and time (T1 and T2) on DNAm. Model 2 included the interaction between group and time to explore group differences in change in DNAm over time. All models controlled for baseline chronological age (grand mean centered at 44.79 years) and sex (0 = male, 1 = female, as a factor variable). Notably, because these statistical models control for level 2 (time-invariant) chronological age and include level 1 (time-varying) time as a predictor, our findings can be considered in terms of “age acceleration”, which in cross-sectional studies is achieved by controlling for chronological age or outputting residuals from DNAm age regressed on chronological age. Sensitivity analyses further controlled for the percentages of six cell subtypes (CD8 T cells, CD4 T cells, NK cells, plasma blasts, monocytes, and granulocytes), treated as time-varying covariates.

Statistical analyses were conducted in R version 4.1.1 using the nlme package (version 3.1.152). The variance-covariance structure was modeled as a random intercept in all models. Gamma weights (γ), analogous to unstandardized beta weights (i.e., a 1-unit change in the predictor [Decliner vs. Maintainer, or T1 vs. T2] is associated with γ-year change in the outcome), are reported with their 95% confidence intervals (CIs) in tables. We adjusted for multiple comparisons using the Benjamini-Hochberg (BH) correction (using the p.adjust function in R) [18]. To examine different levels of stringency, false discovery rates (FDRs) of .05 and .10 were calculated and chosen to ensure no true discoveries were missed while balancing the number of false positives. FDRs can be interpreted as the expected proportion of false positives among all statistically significant tests.

### Power considerations

We selected 24 participants per group to balance funding constraints with generating preliminary data. Although we maximized our ability to detect effects by selecting cognitive groups from extremes of the distribution of change in cognitive performance, the smaller sample size affects our power nonetheless. There is no conventional method for computing power in a multilevel model; however, for a parallel two-group independent *t*-test with 24 participants per group and alpha set to .05, power of 0.80 can detect approximately Cohen’s *d* = 0.82 (see power curve plotted in Supplementary Figure 1). Therefore, the current study was powered to detect large effects for comparing DNAm measures between groups; we had low statistical power to explore group by time interactions on DNAm measures.

## Supplementary Materials

Supplementary Materials

Supplementary Figure 1

Supplementary Tables
