# Supplementary material for: DNA methylation-based measures of biological aging and cognitive decline over 16-years: preliminary longitudinal findings in midlife
Source: Aging (Albany NY). 2022 Nov 11;14(23):9423–44. doi: 10.18632/aging.204376 (PMC9792211; doi:10.18632/aging.204376)
Supplement: Supplementary Materials [file aging-14-204376-s001.pdf]

SUPPLEMENTARY MATERIALS

Introduction

Overview of DNAm clocks

The DNAm clock measures were developed using supervised machine learning techniques to derive algorithms that capture DNAm patterns that predict a dependent variable of interest, or a surrogate of "biological age". The dependent variables differ across the different types of clocks.

First-generation clocks

The first-generation clocks were trained to predict chronological age.

Hannum et al. [1] developed an epigenetic clock (71 CpGs) using whole blood samples from 656 individuals (426 Caucasian and 120 Hispanic) aged 19 to 101. The Hannum clock used in the current study does not include cell distribution data. However, for completeness, there is a version of the Hannum clock known as extrinsic epigenetic age acceleration (EEAA) that is a weighted average of Hannum's estimate with naïve and exhausted CD8 T cells and plasma blasts and adjusted for chronological age [2].

Horvath [3] developed a multi-tissue epigenetic clock (353 CpGs) from 8,000 samples (82 different datasets) representing people across the lifespan. The Horvath clock used in the current study does not include cell distribution data; there is a version of the Horvath clock defined as the residual resulting from regressing Horvath's DNAm age on chronological age and 7 blood cell types (naïve and exhausted CD8 T cells, plasma blasts, CD4 T cells, NK cells, monocytes, and granulocytes) and is known as intrinsic epigenetic age acceleration (IEAA) [4].

Second-generation clocks

The second-generation clocks were optimized for lifespan prediction. Levine et al. [5] proposed the "PhenoAge" clock, which was developed in two steps. First, using data from the National Health and Nutrition Examination Survey (9,926 people ages 20 and over), they developed a measure of "phenotypic age" by selecting from 42 blood-based clinical markers those that predicted mortality. Based on this analysis, 9 blood-based clinical markers (see table below) and chronological age were selected and combined into a phenotypic age estimate and validated in a new sample to predict all-cause mortality. In the second step, data from 465 participants aged 21–100 years in the Invecchiare in Chianti (InCHIANTI) study were used to regress phenotypic age on CpG sites. From this, the PhenoAge clock (513 CpGs) was developed, which strongly relates to all-cause mortality and aging-related morbidity [5].

Phenotypic age	Role
Albumin	Liver
Alkaline phosphatase	Liver
Creatinine	Kidney
Glucose, serum	Metabolic
C-reactive protein	Inflammation
Lymphocyte percent	Immune
Mean (red) cell volume	Immune
Red cell distribution width	Immune
White Blood cell count	Immune

Lu et al. [6] developed the "GrimAge" epigenetic clock in two steps. First, DNAm-based surrogates for self-reported smoking pack-years and a selection of plasma proteins associated with morbidity and mortality were constructed from 2,356 individuals from the Framingham Heart Study offspring cohort (average age: 66 years). Second, time-to-death due to all-cause mortality was regressed on age, sex, DNAm-based pack-years, and 7 DNAm-based surrogate plasma markers (see table below). The resulting mortality risk estimate was transformed into an age estimate, called GrimAge (1030 CpGs).

DNAm based surrogates for plasma proteins	Role
Adrenomedullin	Multiple functions
Beta-2-microglublin	Immune

Cystatin C	Kidney
GDF-15	Stress response
Leptin	Metabolic
Plasminogen activator inhibitor-1 (PAI-1)	Fibrinolytic
Tissue inhibitor matrix metalloproteinase 1 (TIMP-1)	Matrix regulation

Pace of aging measures

Most recently, "pace of aging" measures were developed, which have been referred to as the third-generation of DNAm clocks. Pace of aging measures differ from first- and second-generation clocks in that they are trained to predict *longitudinal* biomarker data. Belsky and colleagues developed the Dunedin PoAm (Pace of Aging from methylation; [7]) and Dunedin PACE (Pace of Aging Calculated from the Epigenome; [8]) measures. Both measures were developed using the Dunedin Study (52% male, 93% white), a longitudinal investigation of individuals born between April 1972 and March 1973 in Dunedin, New Zealand.

The pace of aging measures were developed in two steps, with slight differences highlighted. First, mixed-effects growth curve models were used to estimate longitudinal changes over time in many blood-chemistry and organ-system-function biomarkers across physiological systems (18 biomarkers for Dunedin PoAm; 19 biomarkers for Dunedin PACE – see table below). Biomarkers for Dunedin PoAm were measured across 12 years, at ages 26, 32, and 38. Biomarkers for Dunedin PACE were measured across 20 years, at ages 26, 32, 38, and 45. In other words, these measures were trained in a cohort of same-aged individuals. The slopes were composited across the 18 or 19 biomarkers to calculate a participant's "pace of aging" across 12 years (Dunedin PoAm) or 20 years (Dunedin PACE). Second, elastic-net regression analyses were used to select CpGs that predict the longitudinal pace of aging measures, resulting in Dunedin PoAm (46 CpGs) and Dunedin PACE (173 CpGs). Additional details for developing Dunedin PACE, including the selection of reliable CpG probes, are discussed in Belsky et al. [8].

(Bio)marker	Role	Dunedin PoAm	Dunedin PACE
Glycated hemoglobin (HbA1C)	Metabolic	X	X
Cardiorespiratory fitness (VO_2Max)	Cardiovascular	X	X
Waist-hip ratio	Anthropometric	X	X
Body mass index	Anthropometric	X	X
FEV_1/FVC ratio	Pulmonary	X	X
FEV_1	Pulmonary	X	X
Mean arterial pressure	Cardiovascular	X	X
Leukocyte telomere length	Immune	X	(not included)
Creatinine clearance (eGFR)	Kidney	X	X
Blood urea nitrogen	Kidney	X	X
Triglycerides	Metabolic	X	X
Total cholesterol	Metabolic	X	X
HDL cholesterol	Metabolic	X	X
Lipoprotein *(a)*	Metabolic	X	X
Apolipoprotein B100/A1 ratio	Metabolic	X	X
Gum health (combined attachment loss)	Periodontal	X	X
Caries-affected tooth surfaces	Periodontal	(not included)	X
White blood cell count	Immune	X	X
High-sensitivity C-reactive protein	Inflammation	X	X
Leptin	Metabolic	(not included)	X

Principal components (PC)-based clocks

Traditional epigenetic clocks use individual CpG sites as inputs to the epigenetic age algorithms, but individual CpGs are unreliable and noisy [9]. Therefore, Higgins-Chen et al. proposed [10] that principal components analysis (PCA) can be used to enhance the reliability of traditional epigenetic clocks by extracting shared systematic variation across CpG sites (principal components, PCs) and feeding those PCs into the elastic net regressions to predict chronological age or other health phenotype. Higgins-Chen et al. provides R code that has users project their own DNAm data onto the original PCA space, which then allows PC-based clock outcomes to be estimated from new data. PC-based clocks show agreement between technical replicates (the same sample measured twice) within 0 to 1.5 years and more stable trajectories in longitudinal studies [10]. PC-based clocks have been used in other published studies (e.g., [11]).

Supplementary Results

Normed neuropsychological test scores

The average normed scores for several individual neuropsychological tests at Time 1 and Time 2 are displayed below for each cognitive group (Decliners, Maintainers). The normed scores are represented as T-scores (M[SD] = 50 [10]), with corresponding z-scores and percentile information.

At T1, both cognitive groups had average or slightly above average normed test scores; when averaged across individual tests, Decliners were at the 60th percentile and Maintainers the 62nd percentile. At T2, Decliners were at the 49th percentile whereas Maintainers were at the 73nd percentile.

**Decliners**	**Time 1 (T1)**			**Time 2 (T2)**		
	**T-score**	**z-score**	**Percentile**	**T-score**	**z-score**	**Percentile**
Matrix Reasoning	57.58	0.76	77.6	59.21	0.92	82.2
Digit Span total	57.4	0.74	77.0	51.4	0.14	55.6
Stroop Word	48.79	−0.12	45.2	43.96	−0.60	27.3
Stroop Color	48.25	−0.18	43.1	44.46	−0.55	29
Stroop Color-Word	51.25	0.13	55	49.96	0	49.8
Average	*52.65*	*0.266*	*59.58*	*49.79*	*−0.018*	*48.78*

**Maintainers**	**Time 1 (T1)**			**Time 2 (T2)**		
	**T-score**	**z-score**	**Percentile**	**T-score**	**z-score**	**Percentile**
Matrix Reasoning	58.21	0.82	79.4	63.42	1.3	91.0
Digit Span total	54.3	0.43	66.6	57.2	0.72	76.5
Stroop Word	53.08	0.31	62.1	52.65	0.27	60.5
Stroop Color	51.67	0.17	56.6	52.35	0.24	59.3
Stroop Color-Word	48.88	−0.11	45.5	57.13	0.71	76.2
Average	*53.23*	*0.32*	*62.04*	*56.55*	*0.65*	*72.7*

Supplementary References

1. Hannum G, Guinney J, Zhao L, Zhang L, Hughes G, Sadda S, Klotzle B, Bibikova M, Fan JB, Gao Y, Deconde R, Chen M, Rajapakse I, et al. Genome-wide methylation profiles reveal quantitative views of human aging rates. Mol Cell. 2013; 49:359–67. https://doi.org/10.1016/j.molcel.2012.10.016 PMID:23177740

2. Chen BH, Marioni RE, Colicino E, Peters MJ, Ward-Caviness CK, Tsai PC, Roetker NS, Just AC, Demerath EW, Guan W, Bressler J, Fornage M, Studenski S, et al. DNA methylation-based measures of biological age: meta-analysis predicting time to death. Aging (Albany NY). 2016; 8:1844–65. https://doi.org/10.18632/aging.101020 PMID:27690265

3. Horvath S. DNA methylation age of human tissues and cell types. Genome Biol. 2013; 14:R115. https://doi.org/10.1186/gb-2013-14-10-r115 PMID:24138928

4. Horvath S, Gurven M, Levine ME, Trumble BC, Kaplan H, Allayee H, Ritz BR, Chen B, Lu AT, Rickabaugh TM, Jamieson BD, Sun D, Li S, et al. An epigenetic clock analysis of race/ethnicity, sex, and coronary heart disease. Genome Biol. 2016; 17:171. https://doi.org/10.1186/s13059-016-1030-0 PMID:27511193

5. Levine ME, Lu AT, Quach A, Chen BH, Assimes TL, Bandinelli S, Hou L, Baccarelli AA, Stewart JD, Li Y, Whitsel EA, Wilson JG, Reiner AP, et al. An epigenetic biomarker of aging for lifespan and healthspan. Aging (Albany NY). 2018; 10:573–91. https://doi.org/10.18632/aging.101414 PMID:29676998

6. Lu AT, Quach A, Wilson JG, Reiner AP, Aviv A, Raj K, Hou L, Baccarelli AA, Li Y, Stewart JD, Whitsel EA, Assimes TL, Ferrucci L, Horvath S. DNA methylation GrimAge strongly predicts lifespan and healthspan. Aging (Albany NY). 2019; 11:303–27. https://doi.org/10.18632/aging.101684 PMID:30669119

7. Belsky DW, Caspi A, Arseneault L, Baccarelli A, Corcoran DL, Gao X, Hannon E, Harrington HL, Rasmussen LJ, Houts R, Huffman K, Kraus WE, Kwon D, et al. Quantification of the pace of biological aging in humans through a blood test, the DunedinPoAm DNA methylation algorithm. Elife. 2020; 9:e54870. https://doi.org/10.7554/eLife.54870 PMID:32367804

8. Belsky DW, Caspi A, Corcoran DL, Sugden K, Poulton R, Arseneault L, Baccarelli A, Chamarti K, Gao X, Hannon E, Harrington HL, Houts R, Kothari M, et al. DunedinPACE, a DNA methylation biomarker of the pace of aging. Elife. 2022; 11:e73420. https://doi.org/10.7554/eLife.73420 PMID:35029144

9. Sugden K, Hannon EJ, Arseneault L, Belsky DW, Corcoran DL, Fisher HL, Houts RM, Kandaswamy R, Moffitt TE, Poulton R, Prinz JA, Rasmussen LJH, Williams BS, et al. Patterns of reliability: Assessing the reproducibility and integrity of DNA methylation measurement. Patterns. Elsevier; 2020; 1. Available from: https://www.cell.com/patterns/abstract/S2666-3899(20)30014-3.

10. Higgins-Chen AT, Thrush KL, Wang Y, Minteer CJ, Kuo PL, Wang M, Niimi P, Sturm G, Lin J, Moore AZ, Bandinelli S, Vinkers CH, Vermetten E, et al. A computational solution for bolstering reliability of epigenetic clocks: Implications for clinical trials and longitudinal tracking. Nat Aging. 2022; 2:644–61. https://doi.org/10.1038/s43587-022-00248-2 PMID:36277076

11. Pang APS, Higgins-Chen AT, Comite F, Raica I, Arboleda C, Went H, Mendez T, Schotsaert M, Dwaraka V, Smith R, Levine ME, Ndhlovu LC, Corley MJ. Longitudinal Study of DNA Methylation and Epigenetic Clocks Prior to and Following Test-Confirmed COVID-19 and mRNA Vaccination. Front Genet. 2022; 13:819749. https://doi.org/10.3389/fgene.2022.819749 PMID:35719387
