# Supplementary material for: DNA methylation-based measures of biological aging and cognitive decline over 16-years: preliminary longitudinal findings in midlife
Source: Aging (Albany NY). 2022 Nov 11;14(23):9423–44. doi: 10.18632/aging.204376 (PMC9792211; doi:10.18632/aging.204376)
Supplement: Supplementary Figure 1 [file aging-14-204376-s002.pdf]

SUPPLEMENTARY FIGURE

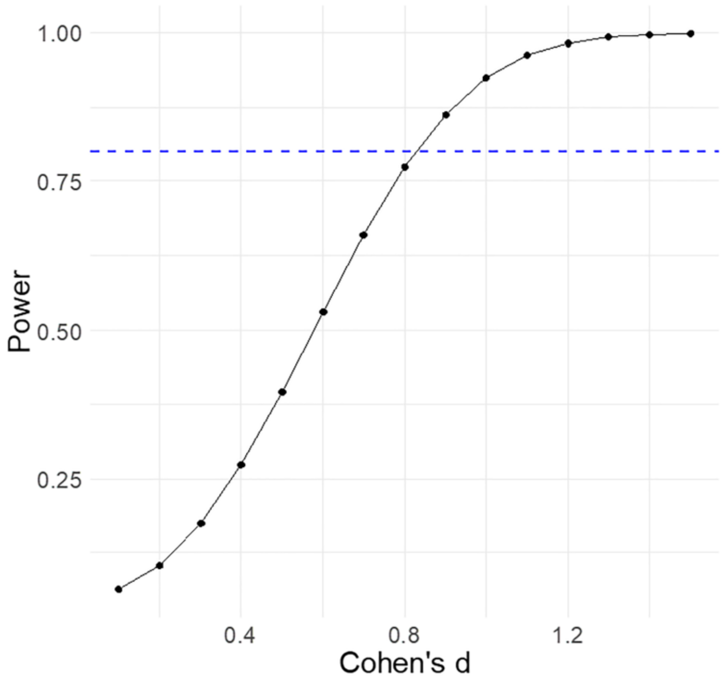

Supplementary Figure 1. Power curve for two-sample independent *t*-test with 24 participants per group and α = .05. Dashed horizontal line indicates power of 0.80.
