# Supplementary material for: DNA methylation-based measures of biological aging and cognitive decline over 16-years: preliminary longitudinal findings in midlife
Source: Aging (Albany NY). 2022 Nov 11;14(23):9423–44. doi: 10.18632/aging.204376 (PMC9792211; doi:10.18632/aging.204376)
Supplement: Supplementary Tables [file aging-14-204376-s003.pdf]

SUPPLEMENTARY TABLES

Supplementary Table 1. Main effects of group and time for traditional epigenetic clocks.

	Horvath		Hannum		PhenoAge		GrimAge	
	γ (CI)	*p*	*γ (CI)*	*p*	*γ (CI)*	*p*	*γ (CI)*	*p*
Intercept	47.40 (45.49– 49.31)	**<0.001**	39.65 (37.98–41.32)	**<0.001**	34.80 (32.47–37.13)	**<0.001**	48.35 (46.06–50.65)	**<0.001**
Female	−0.89 (−2.93–1.16)	0.398	−2.83 (−4.61–−1.05)	**0.003**	−0.58 (−3.02–1.87)	0.646	−1.56 (−4.05–0.93)	0.226
Baseline Age	0.81 (0.65–0.98)	**<0.001**	0.88 (0.74–1.03)	**<0.001**	1.12 (0.93–1.31)	**<0.001**	0.81 (0.61–1.01)	**<0.001**
Group-*Decliners*	−1.17 (−3.19–0.86)	0.265	−1.58 (−3.35–0.19)	0.086	−0.03 (−2.45–2.40)	0.984	1.91 (−0.56–4.38)	0.136
Time	12.75 (11.86–13.64)	**<0.001**	13.11 (12.29–13.93)	**<0.001**	15.30 (13.91–16.70)	**<0.001**	12.42 (11.70–13.15)	**<0.001**
**Random Effects**								
σ^2	4.71		3.99		11.54		3.15	
τ_{00}	9.76 ahabid		7.19 ahabid		11.58 ahabid		16.38 habid	
N	48 ahabid		48 ahabid		48 ahabid		48 ahabid	
Observations	96		96		96		96	

95% Confidence Intervals (CI) are reported.

Supplementary Table 2. Main effects of group and time on PC-clocks and pace of aging measures, controlling for cell percentages.

	PC-Horvath		PC-Hannum		PC-PhenoAge		PC-GrimAge		Dunedin PoAm		Dunedin PACE	
	γ (CI)	*p*	*γ (CI)*	*p*	*γ (CI)*	*p*	*γ (CI)*	*p*	*γ (CI)*	*p*	*γ (CI)*	*p*
Intercept	49.26 (47.71–50.81)	**<0.001**	55.18 (53.63–56.73)	**<0.001**	45.66 (43.39–47.93)	**<0.001**	58.91 (57.14–60.67)	**<0.001**	1.00 (0.96–1.04)	**<0.001**	0.90 (0.84–0.96)	**<0.001**
Female	−3.06 (−4.70–−1.41)	**0.001**	−2.51 (−4.16–−0.87)	**0.006**	−1.83 (−4.28–0.61)	0.163	−2.09 (−4.01–−0.17)	**0.045**	0.01 (-0.03–0.05)	0.742	−0.01 (−0.07–0.06)	0.882
Baseline Age	0.84 (0.71–0.97)	**<0.001**	0.86 (0.73–0.99)	**<0.001**	1.06 (0.86–1.25)	**<0.001**	0.79 (0.64–0.94)	**<0.001**	0.00 (-0.00–0.00)	0.399	0.00 (−0.00–0.01)	0.677
Group-*Decliners*	−0.59 (−2.23–1.04)	0.495	−0.22 (−1.85–1.41)	0.799	1.66 (−0.77–4.09)	0.201	2.15 (0.24–4.05)	**0.038**	0.04 (0.00–0.09)	**0.048**	0.08 (0.01–0.14)	**0.030**
Time	11.25 (10.58–11.91)	**<0.001**	11.74 (11.07–12.41)	**<0.001**	13.73 (12.97–14.48)	**<0.001**	12.18 (11.74–12.61)	**<0.001**	0.02 (0.01–0.04)	**0.012**	0.03 (0.00–0.06)	**0.030**
CD8 T cell	−11.78 (−94.81–71.25)	0.789	−14.70 (−98.24–68.84)	0.740	67.84 (−27.49–163.18)	0.184	−10.84 (−66.86–45.17)	0.715	−1.83 (−3.70–0.03)	0.069	−0.59 (−3.90–2.72)	0.738
CD4 T cell	6.63 (−36.09–49.34)	0.770	19.75 (−23.22–62.73)	0.388	48.93 (−0.11–97.98)	0.065	21.35 (−7.47–50.16)	0.167	−0.67 (−1.63–0.29)	0.192	0.28 (−1.43–1.98)	0.760
NK cell	30.17 (−17.47–77.80)	0.236	49.66 (1.73–97.58)	0.056	78.80 (24.13–133.47)	**0.009**	23.22 (−8.89–55.34)	0.177	−0.20 (−1.27–0.87)	0.720	0.16 (−1.74–2.06)	0.873
Plasma Blasts	0.28 (−5.08–5.64)	0.921	1.47 (−3.92–6.86)	0.606	0.36 (−5.81–6.54)	0.912	0.55 (−3.09–4.18)	0.776	−0.10 (−0.22–0.03)	0.141	0.03 (−0.19–0.24)	0.819
Monocytes	6.43 (−47.64–60.50)	0.822	28.13 (−26.27–82.53)	0.332	70.73 (8.56–132.89)	**0.037**	27.31 (−9.25–63.87)	0.163	−0.74 (−1.96–0.47)	0.252	−0.30 (−2.45–1.86)	0.795
Granulocytes	19.49 (−17.07–56.05)	0.317	35.31 (−1.48–72.09)	0.075	80.82 (38.88–122.76)	**0.001**	33.02 (8.40–57.65)	**0.015**	−0.09 (−0.91–0.73)	0.836	0.53 (−0.93–1.99)	0.494
**Random Effects**												
σ^2	1.97		1.99		2.48		0.84		0.00		0.00	
τ_{00}	6.75 ahabid		6.69 ahabid		15.96 ahabid		10.22 ahabid		0.00 ahabid		0.01 ahabid	
N	48 ahabid		48 ahabid		48 ahabid		48 ahabid		48 ahabid		48 ahabid	
Obs	96		96		96		96		96		96	

95% Confidence Intervals (CI) are reported.

Supplementary Table 3. Scaled cognitive components for decliners (*n* = 24) and maintainers (*n* = 24).

Scaled cognitive component	*n* miss[a]	Overall	Decliners	Maintainers	*p*-value[b]
Matrix Reasoning					
T1	0	74.31 (12.21)	74.23 (10.04)	74.38 (14.28)	0.966
T2	0	70.45 (13.93)	65.59 (15.81)	75.31 (9.87)	**0.014**
Digit Span – Forward					
T1	0	52.84 (24.93)	56.44 (21.94)	49.24 (27.59)	0.322
T2	0	50.19 (21.22)	43.56 (19.14)	56.82 (21.49)	**0.029**
Digit Span – Backwards					
T1	0	53.82 (24.31)	56.25 (24.97)	51.39 (23.91)	0.494
T2	0	44.44 (19.85)	40.63 (15.98)	48.26 (22.79)	0.185
Trail Making Test (A)					
T1	0	95.86 (2.70)	95.19 (2.90)	96.52 (2.37)	0.087
T2	0	94.35 (4.90)	92.36 (5.97)	96.34 (2.28)	0.004
Trail Making Test (A-B)					
T1	0	87.18 (9.52)	86.80 (10.80)	87.57 (8.26)	**0.784**
T2	0	84.28 (12.45)	80.82 (15.13)	87.73 (7.94)	0.053
Stroop Word					
T1	0	53.19 (15.60)	49.67 (16.37)	56.70 (14.27)	0.12
T2	1	43.83 (16.42)	35.69 (15.47)	52.33 (12.88)	**<0.001**
Stroop Color					
T1	0	57.19 (14.90)	54.61 (14.59)	59.77 (15.07)	0.234
T2	1	50.08 (17.17)	42.46 (16.25)	58.02 (14.52)	**0.001**
Stroop Color-Word					
T1	0	38.24 (15.86)	40.63 (17.53)	35.85 (13.96)	0.302
T2	1	36.14 (17.40)	29.47 (14.70)	43.09 (17.55)	**0.006**
Digit Vigilance – page 1					
T1	0	79.20 (12.61)	81.17 (11.04)	77.23 (13.96)	0.284
T2	19	71.42 (19.84)	50.24 (24.60)	79.49 (9.59)	**<0.001**
Digit Vigilance – page 2					
T1	0	78.65 (16.08)	81.15 (11.44)	76.16 (19.61)	0.287
T2	20	72.18 (17.50)	53.70 (18.13)	78.34 (12.46)	**<0.001**

Means and standard deviations (SD, in parentheses) are displayed for all scaled cognitive measures (scaled using the proportion of maximum scaling method, range: 0-100, see Methods). Higher scores indicate better performance. T1 = time 1; T2 = time 2. [a]At T1, no cognition data were missing. At T2, 1 participant was missing the Stroop test and 19 were missing Digit Vigilance pages 1 and 2 and 1 was missing just page 2. [b]*p*-value comparing groups using dependent *t*-tests. *p*-values are bold if <0.05.

Supplementary Table 4. Main effects of scaled T2 cognitive components on PC-GrimAge and pace of aging measures, controlling for cell percentages.

	PC-GrimAge		Dunedin PoAm		Dunedin PACE	
	γ (CI)	*p*	*γ (CI)*	*T2 Predictors*	*γ (CI)*	*p*
Matrix Reasoning	−0.066 (−0.138–0.005)	0.086	−0.001 (−0.003–0.001)	0.255	−0.003 (−0.005–−0.001)	**0.021**
DS-Forward	−0.024 (−0.074–0.025)	0.356	−0.000 (−0.001–0.001)	0.794	−0.001 (−0.003–0.001)	0.278
DS-Backward	−0.034 (−0.086–0.019)	0.229	−0.000 (−0.001–0.001)	0.602	−0.001 (−0.003–0.001)	0.277
Trail A	−0.177 (−0.385–0.030)	0.113	−0.004 (−0.008–0.001)	0.140	−0.008 (−0.015–−0.001)	**0.045**
Trail A-B	−0.110 (−0.184–−0.035)	**0.008**	−0.002 (−0.004–−0.001)	**0.012**	−0.004 (−0.006–−0.001)	**0.012**
Stroop Word	−0.059 (−0.119–0.002)	0.074	−0.001 (−0.002–0.000)	0.116	−0.002 (−0.005–−0.000)	**0.038**
Stroop Color	−0.060 (−0.120–0.000)	0.066	−0.001 (−0.002–0.000)	0.087	−0.001 (−0.004–0.001)	0.225
Stroop Color-Word	−0.079 (−0.133–−0.024)	**0.009**	−0.001 (−0.002–0.000)	0.097	−0.002 (−0.004–0.000)	0.087
Digit Vigilance-pg1	−0.056 (−0.131–0.018)	0.173	−0.001 (−0.003–0.000)	0.165	−0.001 (−0.004–0.002)	0.466
Digit Vigilance-pg2	−0.068 (−0.146–0.010)	0.122	−0.002 (−0.004–0.000)	0.123	−0.002 (−0.005–0.001)	0.227

95% Confidence Intervals (CI) are reported. Models included female, baseline age, time, and cell percentages (estimates not shown). Higher scaled cognitive scores indicate better performance. Abbreviation: DS: digit span.
